# The King's Illness

**Published:** 1902-07-05

**Authors:** 


### The Hospital

A Journal of

The Medical Sciences and Hospital Administration.

Vol. XXXII.—No. 823. Edited by Sir Henry Burdett, K.C.B., and Solomon C. Smith, M.D., M.R.C.P. [July 5, 1902.

### The King's Illness.

The events of the King's illness, during the last week, have constituted an episode of history in the presence of which all contemporary occurrences have sunk into insignificance. Notwithstanding the reserve which habitually softens the "fierce light which beats upon a throne" many people had come to hear or to suspect, even towards the close of the week preceding that fixed for the great ceremony, that the health of the august central figure therein was by no means satisfactory. Most men are so constituted as to assign undue prominence to evidence which points towards the fulfilment of their cherished hopes, and comparatively to disregard whatever may point in an opposite direction. In the case of His Majesty and of his advisers, at any rate, it seems certain that the optimistic view prevailed over the pessimistic; and, whatever may be said by those who are wise after the event, it would be both idle and unfair to suggest that the former was one which, in all the circumstances, should have been rejected. We must be content to know that, on whatever grounds it was supposed to rest, it was not borne out by events; that on Monday evening grave symptoms appeared, and, in the course of the next twelve hours, so far increased as to leave no hope that His Majesty could go through the Coronation, or even that a further period of waiting could be permitted without great risk to his life. "Perityphlitis" was declared to be present in a serious form, and an operation to be imperatively necessary. The King is reported to have been ready to incur any danger if he could by any possibility avoid inflicting a disappointment upon his people, but his professional advisers had the courage and the wisdom to insist; and His Majesty recognised the truth of the proverb that "the sick man's chamber is the kingdom of the physician."

Concerning the precise character of the conditions disclosed by the operation, we have as yet only been told that an abscess was found and opened, and that a considerable quantity of pus was removed. It may probably be assumed that the appendix was removed also, although this has not been stated. But the bulletins have pointed to two distinct possibilities of danger; first, from the absorption of septic material; secondly, from imperfect union of a solution of continuity of the intestinal wall. The former may be regarded as that which is now declared to have been obviated; the latter as one about which every hope may reasonably be entertained and encouraged, but as to which there cannot at present be absolute certainty. Even while we write, a stage has been reached at which it may almost be said that there is no longer any danger; but there are unlikely contingencies from which danger might possibly arise. Before these lines come into the hands of our readers, we trust that these contingencies will have been set aside by the course of events, and that we may look forward to a speedy and uninterrupted convalescence.

Of the events to which that convalescence may lead it would at present be premature to speak with any certainty; but the hope is entertained in many quarters that the Coronation ceremony will not be long delayed and that the King will wait until after its performance before he seeks the period of rest which will no doubt be suggested as a necessity for his complete restoration to health and vigour. For this arrangement there is much to be said, not the least cogent of the reasons in favour of its adoption being that the Coronation and all its attendant fatigues and worries would be over and done with, and that, when this was so, the cares of State might more easily be reduced to a minimum. In such circumstances, no doubt, the great ceremony would be curtailed of the magnificence which it was originally designed to possess, and would be rendered as little fatiguing as possible; but any changes productive of such results would not be wholly undesirable. It would probably be impossible, at a later period of the year, and after one heavy national disappointment, to organise such a representation of the public and of the empire as would have greeted the sovereigns on the 26th and 27th of June; and, in all the circumstances, the expediency would be at least as doubtful as the possibility. In the meanwhile, the King has received from his people such a tribute of loyalty and affection as monarch has never received before. Money probably to be reckoned by millions has been spent, enormous losses have been incurred in the conduct of what appeared to be legitimate commercial enterprise, and thousands of people from all parts of the world have sustained bitter disappointment; but all these accumulated troubles have been powerless to call forth a complaining note. The danger to the King has effaced the sense of personal grievance and of personal loss, and the improvement of the King has converted a calamity into an occasion for rejoicing. Surely no jewel of equal value has ever before adorned a crown.

